# Comparison of the Ventricle Muscle Proteome between Patients with Rheumatic Heart Disease and Controls with Mitral Valve Prolapse: HSP 60 May Be a Specific Protein in RHD

**DOI:** 10.1155/2014/151726

**Published:** 2014-03-12

**Authors:** Dawei Zheng, Limin Xu, Lebo Sun, Qiang Feng, Zishan Wang, Guofeng Shao, Yiming Ni

**Affiliations:** ^1^Department of Cardiothoracic Surgery, The Affiliated Hospital, Ningbo Medical Centre Lihuili Hospital, Ningbo University, Ningbo, Zhejiang 315041, China; ^2^Department of Cardiothoracic Surgery, The First Affiliated Hospital, School of Medicine, Zhejiang University, Hangzhou, Zhejiang 310006, China

## Abstract

*Objective*. Rheumatic heart disease (RHD) is a serious autoimmune heart disease. The present study was aimed at identifying the differentially expressed proteins between patients with RHD and controls with mitral valve prolapse. *Methods*. Nine patients with RHD and nine controls with mitral valve prolapsed were enrolled for this study. Two-dimensional difference in-gel electrophoresis (2D-DIGE) and matrix-assisted laser desorption ionization time-of-flight mass spectrometry (MALDI-TOF-MS) were performed. *Results*. A total of 39 protein spots with differential expressions were identified between the two groups (*P* < 0.05, Average Ratio > 1.2 or Average Ratio < −1.2) and four upregulated proteins (including heat shock protein 60 (HSP 60), desmin, PDZ and LIM domain protein 1, and proteasome subunit alpha type-1) and three downregulated proteins (including tropomyosin alpha-1 chain, malate dehydrogenase, and chaperone activity of bc1 complex homolog) were determined. *Conclusion*. These seven proteins, especially HSP 60, may serve as potential biomarkers for the diagnosis of RHD and provide evidence to explain the mechanisms of this complex disease in the future.

## 1. Introduction

Autoimmunity is the failure of an organism to recognize its own constituent parts as self, thus leading to an immune response against its own cells and tissues. Rheumatic heart disease (RHD) is primarily autoimmune sequelae of acute rheumatic fever (ARF) [1, 2], which occurs after group A beta-hemolytic streptococcal pharyngeal infection [3]. RHD can cause chronic inflammation of the endocardium and myocardium, leading to valvular dysfunction and hemodynamic changes and ultimately resulting in heart failure or stroke and other serious consequences. Due to the lack of a specific means of detection of RHD, many patients have been diagnosed with irreversible valvular dysfunction and scheduled for valvular surgery. RHD continues to be a burden in several developing countries such as India and China, although in the western countries it is reasonably rare probably due to the widespread use of antibiotics [1, 4, 5]. Therefore, an ideal biomarker that can represent the characteristic pathophysiological process of RHD will be valuable for the early diagnosis of this disease, which will help patients avoid surgery by early and effective drug therapy.

Proteomics is the largescale study of proteins, particularly their structures and functions, which enables detection and identification of low-abundance proteins. Proteomics has been extensively used to screen diagnostic biomarkers of diseases such as breast cancer and coliform mastitis [6, 7]. Only one proteomics study of the valvular tissue with RHD was performed [8]. However, no study of myocardium with RHD has been performed earlier.

In this study, two-dimensional differential in-gel electrophoresis (2D-DIGE) and matrix-assisted laser desorption ionization time-of-flight mass spectrometry (MALDI-TOF-MS) were used to identify the differentially expressed proteins of myocardium in the RHD and the mitral valve prolapse groups. The present study was aimed at identifying the biomarkers, detecting the chronic inflammatory myocardium injury after ARF, and exploring their significance and mechanism in the pathophysiological process of the myocardial lesion in RHD.

## 2. Materials and Methods

### 2.1. Sample Collection

The inclusion criteria for the experimental group are as follows: (i) every patient diagnosed as rheumatic mitral valve insufficiency with or without mitral stenosis and scheduled for mitral valve replacement; (ii) normal preoperative erythrocyte sedimentation rate and antistreptolysin O to eliminate rheumatism in active stage; (iii) all patients in New York Heart Association (NYHA) functional class II-III; and (iv) no other complications; the patients with acute heart failure were excluded. The inclusion criteria of the control group are as follows: (i) every patient diagnosed as mitral valve prolapse because of mitral chordae tendineae fracture and mitral insufficiency and scheduled for mitral valve replacement. The other conditions are the same as criteria (iii)–(v) of the experimental group. RHD cases and their controls were well matched based on the following details: (i) same gender, (ii) difference of age < 5 years old, (iii) difference of left ventricular ejection fraction (EF) < 5%, (iv) difference of left ventricular end-diastolic diameter (LVEDD) < 10% of the larger of the two; and (v) other physiological indexes from physical check in close. Left ventricular papillary muscle was transferred from resected mitral valve to physiological saline and liquid nitrogen and then moved to −80°C refrigerator for storage. Three male and six female pairs totally matched according to the matching principle. The characteristics of pairing groups were presented in Table 1. The study protocol was approved by the Ethics Committee of Ningbo Lihuili Hospital, and informed consent was obtained from all the subjects.

### 2.2. Sample Preparation for 2D-DIGE

DIGE lysis buffer was added and ground upon ice. The samples were centrifuged and the supernatant was collected to detect the protein concentration. The samples were diluted to 5 *μ*g/*μ*L and the 18 samples were mixed with equal quantity, like 50 *μ*g mixture contains 2.78 *μ*g per sample. The mixture was subpackaged for 50 *μ*g per 10 *μ*L as the internal standard. The pH of the sample was adjusted to 8.0-9.0 for further dye marking. Fifty *μ*g of each sample was labeled with fluorescent dye (GE Healthcare) (the internal standard was labeled Cy2, the experimental group sample was labeled Cy3, and the control group sample was labeled Cy5). The proteins were placed on ice in the dark for 30 minutes for labeling, and finally lysine was added to terminate the reaction.

### 2.3. ****2D-DIGE

The marked samples were combined and sample buffer was added on the ice for 10 minutes. The hydration buffer was added into the labeled samples to a total volume of 250 *μ*L for hydrating the immobilized pH gradient (IPG) strips (pH 3–10 NL, GE Healthcare), followed by isoelectric focusing (IEF). After IEF, place the IPG strips in the equilibration buffer A and equilibration buffer B, in turn, to reduce the disulfide bonds. For the second dimension, the IPG strips were placed on 12.5% polyacrylamide gels for sodium dodecyl sulfate polyacrylamide gel electrophoresis.

### 2.4. Images Scanning and Analysis

The individual images of Cy2-, Cy3-, and Cy5-labeled proteins of each gel were obtained using Typhoon FLA9000 imager (GE Healthcare) with the wavelengths of 488 nm (Cy2), 532 nm (Cy3), and 633 nm (Cy5), respectively. The analysis of images was performed through DeCyder 6.5 software (GE Healthcare) to identify the different expression levels of proteins displayed. *t*-test *P* value and Average Ratio (control group/experimental group) were used to select differentially expressed protein spots. Protein expression value with an Average Ratio > 1.2 or Average Ratio < −1.2 and *P* < 0.05 was considered to be statistically significant.

### 2.5. Protein Identification

All the protein spots of interest were selected and excised manually. Sequencing-grade trypsin (Promega, USA) was added for digestion overnight at 37°C and the enzymatic hydrolysate was collected. ZipTip (Millipore, USA) desalination was performed.

The samples were mixed with alpha-cyano-4-hydroxycinnamic acid (HCCA) matrix as a 1 : 1 relationship. The MS and MS/MS data for protein identification were obtained through 4800 Plus MALDI TOF/TOFTM Analyzer (Applied Biosystems). Combined peptide mass fingerprinting and MS/MS queries were performed using the MASCOT search engine 2.2 (Matrix Science, Ltd) embedded into GPS-Explorer Software 3.6 (Applied Biosystems) on the National Center for Biotechnology Information database.

## 3. Results

**Figure 1:**
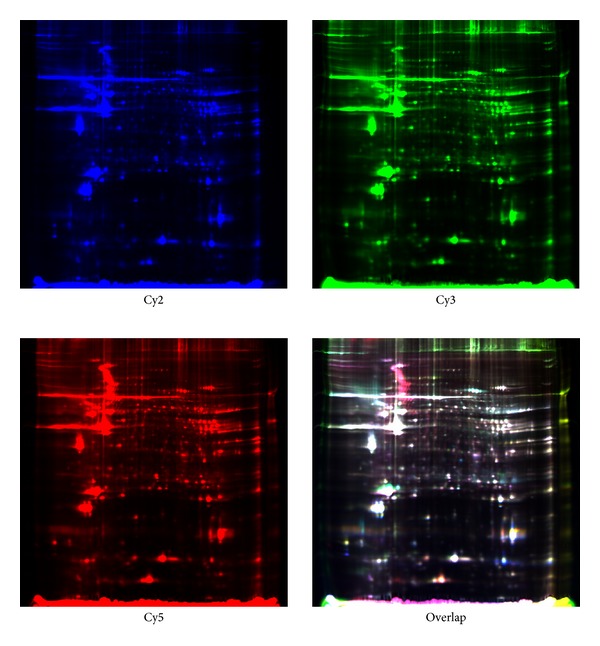
The 2D-DIGE images of left ventricular papillary muscle protein. Internal control samples containing the proteins of experimental group and control group labeled with Cy2 (blue). Proteins of experimental group were labeled with Cy3 (green) while proteins of control group were labeled with Cy5 (red). Color picture was the overlapping images.

**Figure 2:**
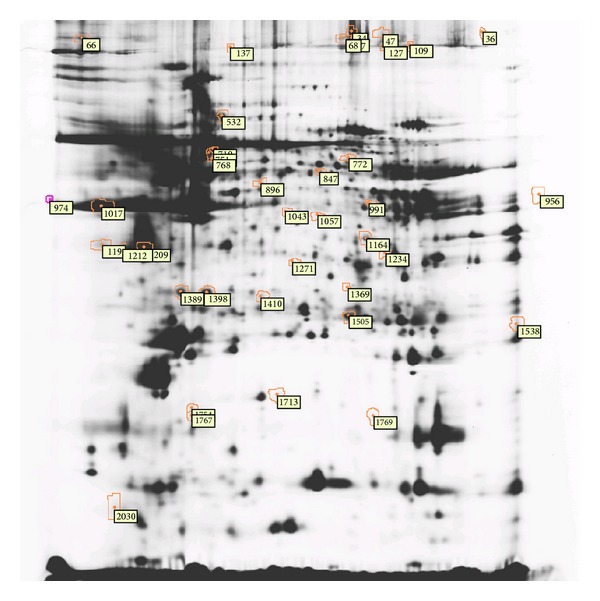
The differential protein spots of RHD left ventricular papillary muscle labeled with number.

2D-DIGE was performed for the nine pairs and 27 maps of 2D gel were obtained (nine maps each for internal standard, experimental group, and control group, resp.). The DIGE images of the left ventricular papillary muscle protein were presented in Figure 1. The distribution and relative intensity of protein spots between groups were consistent. The protein spots in images of RHD mitral valve lesions were compared with those of mitral valve prolapse and 39 differentially expressed proteins were identified as the criterion that *t*-test *P* value < 0.05, Average Ratio > 1.2, or Average Ratio < −1.2. The spots with differential expressions were numbered in Figure 2 and their information was presented in Table 2. Of these, 18 spots were overexpressed more in the RHD group than in the control group (Average Ratio < −1.2) and the remaining 21 spots were expressed stronger in the control group (Average Ratio > 1.2).

MALDI-TOF-MS instruments are ideal for protein identification and also for enabling the identification of several proteins in one spot if they are not separated in the electrophoretic procedure. After the incision and enzymolysis of the 28 special spots (failed to identify the other 11 spots) (Table 3), the MALDI-TOF-MS was used to analyze the differential proteins. Finally, 16 spots were successfully identified. The criterion of successful identification was the protein score CI > 95%, while the protein score was > 50. The results were shown in Table 4. There were 10 proteins overexpressed in the experimental group and six proteins overexpressed in the control group. The heat shock protein 60 (HSP 60) (Average Ratio = −3.17) level was more than three times upregulated in the experimental group. With the alpha-actin presenting an equivocal result, more than one alpha-actin was upregulated or downregulated in the experimental group (Average Ratio = −1.92, 3.37, 1.23, −1.39, and 1.4).

## 4. Discussion

Patients with RHD who were scheduled to undergo mitral valve replacement were selected as the experimental group, and patients with mitral valve prolapse were selected as the control group. The two groups were matched in gender, age, EF, and LVEDD to get an exact contrast. Similar EF and LVEDD can eliminate the difference caused by other associated factors such as heart failure. Those proteins whose Average Ratio > 1.5 or < −1.5 were considered to be statistically significant in the difference of protein expression. As a result, 11 differentially expressed proteins were identified. There are seven structure proteins (four types of alpha-actin, desmin, tropomyosin alpha-1 chain, and PDZ and LIM domain protein 1), two zymoproteins (malate dehydrogenase (MDH) and proteasome subunit alpha type-1), and two molecular chaperones (HSP 60 and chaperone activity of bc1 complex homolog (CABC1) protein). The actins were identified in more than one spot due to the great richness of actins in the cardiac muscle. There were four proteins overexpressed in the experimental group (HSP 60, desmin, PDZ and LIM domain protein 1, and proteasome subunit alpha type-1) and three proteins overexpressed in the control group (tropomyosin alpha-1 chain, MDH, and CABC1 protein).

Heat shock proteins (HSPs) are a family of highly conserved, protective proteins expressed in all cells. They primarily protect cells by folding denatured proteins, stabilizing macromolecules, and targeting irreversibly denatured proteins for clearance [9]. However, some findings implied that the released HSP 60 can have a toxic effect on the surrounding cardiac myocytes and lead to apoptosis when myocardium is injured [10, 11]. Extracellular HSP may participate in the inflammatory and autoimmune disorders by activating the innate immune response [12, 13]. As a ligand of toll-like receptor- (TLR-) 4, extracellular HSP 60 can activate TLR-4, which could cause cardiac myocyte apoptosis and inflammatory cytokine production [10, 11, 14]. Intracellular HSP 60 was released into the media, which also caused cytokine production and TLR-4 overexpression [14]. Although the resected papillary muscle was removed from the serum and pericardium and treated with physiological saline, it was quite difficult to confirm whether the HSP 60 changes detected were extracellular or intracellular considering the innate nature of RHD. It was found that HSP 60 was significantly increased in patients with RHD (3.17 times higher than the control group). However, the overexpression of HSP 60 can activate TLR-4 and potentially stimulate the immune diseases. Although other studies have suggested that HSP 60 levels were increased in the failing heart [15, 16] and the ischemia-reperfusion cardiac muscle [17], the influence of congestive heart failure and ischemia-reperfusion injury has been eliminated in the design of the present study. The unique research about acute RHD and HSP family protein suggested that the HSP 60, HSP 73, and HSP 78 were associated with RHD and the autoimmunity process [18]. It is worthy to observe that in the research the sera from patients with acute RHD were collected as the study sample, while the left ventricular papillary muscle was collected from patients with chronic RHD. The present research indicates the role that HSP 60 plays in myocardial impaired process in RHD more intuitively than sera. Lin and colleagues identified HSP 60 on the surface of cardiac myocytes from failing hearts and suggested that the increased HSP 60 may be deleterious [15]. Other researchers suggested that the increase of HSP 60 may be driven by transcription factor nuclear factor-kappaB (NF-*κ*B) activation [19, 20]. The activated NF-*κ*B can contribute to the immune reaction [21], while the proteasome inhibitor can inhibit the activity of NF-*κ*B [21, 22]. Proteasome participated in the synthesis of active NF-*κ*B. The increased HSP expression can label the misfolding and unfolding proteins for degradation by proteasome [19]. In the present research, the two proteins were both increased in experimental group (3.17 times for HSP 60 and 1.71 times for proteasome subunit alpha type-1). Thus, as the influence of heart failure and ischemia-reperfusion injury has been eliminated, there is a belief that the interaction of HSPs and proteasome may play an important role in apoptosis and inflammation reaction in the myocardium with RHD. This also leads to the inference that the upregulated HSP 60 may be a biomarker for RHD, but certainly further research is required.

Desmin is one of the critical cytoskeleton proteins of cardiomyocytes that will increase due to the myocardial hypertrophy in patients with heart failure [23, 24]. Another two studies pointed out that the myocardial tissue of patients with end-stage heart failure revealed a decrease in or lack of desmin expression [25, 26]. According to Monreal et al. [27], increased desmin expression seems to be a sensitive marker of an early cellular response to mechanical stretch, while the decreased or lack of desmin expression may usually happen in the end stage of some serious cardiac diseases, such as heart failure and idiopathic dilated cardiomyopathy. In the present study, the desmin expression in the experimental group is 1.62 times compared with that in the control group, which we conjectured is because of the longer course of disease and the more significant myocardial hypertrophy in patients with RHD.

PDZ and LIM domains containing proteins play diverse biological roles, such as regulation of actin structure, and have been implicated in cardiac and skeletal muscle structure, function, and disease [28–31]. The actinin-associated LIM protein (ALP) subfamily proteins are expressed at the highest levels in skeletal and cardiac muscle [32]. Mouse models and* in vitro* studies suggested that ALP deficiency may influence the development of the right ventricle and ALP enhances the ability of *α*-actinin to cross-link actin filaments [33–35]. The overexpression of PDZ and LIM domain protein 1 (Average Ratio = −1.53, *P* = 0.034) in the present research may play the role of ALP, which interacts with the *α*-actinin and enhances the function of actin filament.

Actin and tropomyosin are major components of the actin microfilament system [36]. Tropomyosin is widely distributed in all cell types along the length of actin filaments [37, 38] and regulates the rates of cardiac contraction and relaxation with actin and the troponin complex [39]. In the present research, the decreased expression of tropomyosin *α*-1 chain (also called *α*-tropomyosin) in the experimental group (Average Ratio = 1.34, *P* = 0.0044) may influence the relaxation and contraction rate of heart.

MDH catalyzes the conversion of oxaloacetate and malate [40]. The activity of cytoplasmic MDH was decreased with senescence due to shortening of telomere length [41, 42]. It has been reported that cytoplasmic MDH family was significantly decreased in patients with dilated cardiomyopathy by 2D-DIGE [43]. CABC1 is a mitochondrial protein similar to yeast CABC1. The* CABC1* gene, also called* CoQ8* or* ADCK3*, is one of the genes involved in the ubiquinone biosynthesis pathway. A group of* CABC1* gene mutations (R213W, G272V, G272D, and E551K) were identified in ubiquinone-deficient patients with familiar neurologic disease, which caused respiratory-chain impairment and ubiquinone deficiency in muscle tissue [44, 45]. Inhibiting the* CABC1* gene expression partially suppresses p53-induced apoptosis [46]. The association between CABC1 and cardiac diseases was not found. In the present research, the MDH and CABC1 proteins decreased in the RHD group (Average Ratio = 1.61, *P* = 0.032, Average Ratio = 1.59, *P* = 0.0045, resp.). The development of disease may influence the metabolism and cellular processes [47].

The 2D-DIGE experiment is based on fluorescence-based quantitation and the low-sensitivity poststaining may influence the detection. Therefore, numerous low-abundance but differentially expressed dye-labeled proteins may be failed to be imaged. A total of 39 differentially expressed proteins were identified by 2D-DIGE. There were 11 differential spots that failed to be identified from gel incision. The reasons may be as follows: (i) a portion of the low-abundance proteins were covered owing to the different sampling amounts; (ii) the different coloration methods for proteins caused the difference; and (iii) the samples degraded. Finally, 16 of the 28 spots were successfully identified for which MALDI-TOF-MS experiment was performed. A further western bolt experiment was impossible to perform at this point in time due to the lack of samples from patients with RHD; however, it is known that a confirmed experiment is necessary.

## 5. Conclusion

In conclusion, in this study, there are seven special proteins found to be significantly different in abundance between the patients with RHD and controls detected through the 2D-DIGE and MALDI-TOF-MS methods. Four proteins, namely, HSP60, desmin, PDZ and LIM domain protein 1, and proteasome subunit alpha type-1, were increased in the experimental group, whereas the other three proteins, namely, tropomyosin alpha-1 chain, MDH, and CABC1 protein, were decreased in the experimental group. HSP 60 may play an important role in the autoimmune pathological process of RHD and could be regarded as a biomarker for RHD. However, this hypothesis needs further confirmation.

## Figures and Tables

**Table 1:** Information of the experimental group and control group.

Group number	Samples	Gender	Age	EF	LVEDD (mm)	Mitral valve stenosis
1	Experimental group	Male	51	0.6	48	Yes
	Control group	Male	51	0.56	54	No
2	Experimental group	Female	49	0.61	45	Yes
	Control group	Female	46	0.59	50	No
3	Experimental group	Female	33	0.68	55	Yes
	Control group	Female	38	0.65	60	No
4	Experimental group	Male	54	0.53	60	No
	Control group	Male	57	0.55	62	No
5	Experimental group	Male	25	0.66	49	Yes
	Control group	Male	30	0.66	47	No
6	Experimental group	Female	21	0.66	43	Yes
	Control group	Female	18	0.63	48	No
7	Experimental group	Female	55	0.56	63	Yes
	Control group	Female	57	0.53	60	No
8	Experimental group	Female	42	0.51	65	Yes
	Control group	Female	47	0.5	67	No
9	Experimental group	Female	43	0.49	55	Yes
	Control group	Female	47	0.51	60	No

**Table 2:** Statistical analysis of abundance difference in differential protein spots (Average Ratio: control group/experimental group).

Number	Spot code	*t*-test *P* value	Average ratio
1	34	0.011	1.46
2	36	0.047	1.77
3	47	0.011	1.58
4	57	0.019	1.52
5	66	0.032	1.36
6	68	0.046	1.52
7	109	0.012	1.95
8	127	0.026	1.59
9	137	0.035	1.67
10	532	0.022	−3.66
11	716	0.042	−3.17
12	751	0.00079	−1.92
13	768	0.033	−1.62
14	772	0.021	−1.28
15	847	0.048	−1.53
16	896	0.035	−1.94
17	956	0.0063	1.4
18	974	0.0054	3.37
19	991	0.043	−1.4
20	1017	0.048	1.23
21	1043	0.001	−1.39
22	1057	0.045	−1.38
23	1164	0.034	−1.53
24	1198	0.013	1.24
25	1209	0.0044	1.34
26	1212	0.011	1.24
27	1234	0.032	1.61
28	1271	0.01	−1.77
29	1369	0.017	−1.71
30	1389	0.016	1.36
31	1398	0.023	1.21
32	1410	0.025	−1.52
33	1505	0.048	−1.2
34	1538	0.042	−1.33
35	1713	0.018	−1.39
36	1754	0.0045	1.59
37	1767	0.025	1.31
38	1769	0.048	−1.59
39	2030	0.0089	1.57

**Table 3:** Information of differential protein spots through MALDI-TOF-MS analysis.

Number	Spot code	Target number	*t*-test *P* value	Average ratio	Protein name
1	532	I2	0.022	−3.66	Failed
2	751	I3	0.00079	−1.92	Alpha-actin
3	716	I4	0.042	−3.17	Heat shock protein 60
4	768	I5	0.033	−1.62	Desmin
5	772	I6	0.021	−1.28	Failed
6	847	I7	0.048	−1.53	Failed
7	896	I8	0.035	−1.94	Failed
8	974	I9	0.0054	3.37	Alpha-actin
9	1017	I10	0.048	1.23	Alpha-actin
10	1043	I11	0.001	−1.39	Alpha-actin
11	1057	I12	0.045	−1.38	Failed
12	991	I13	0.043	−1.4	Elongation factor Tu
13	956	I14	0.0063	1.4	Alpha-actin
14	1212	I15	0.011	1.24	Failed
15	1209	I16	0.0044	1.34	Tropomyosin alpha-1 chain
16	1164	I17	0.034	−1.53	PDZ and LIM domain protein 1
17	1234	I18	0.032	1.61	Malate dehydrogenase
18	1271	I19	0.01	−1.77	Failed
19	1369	I20	0.017	−1.71	Proteasome subunit alpha type 1
20	1389	I21	0.016	1.36	Failed
21	1398	I22	0.023	1.21	Failed
22	1410	I23	0.025	−1.52	Failed
23	1505	I24	0.048	−1.2	Peroxiredoxin 6
24	1538	J1	0.042	−1.33	Cysteine and glycine-rich protein 3
25	1754	J2	0.0045	1.59	CABC1 protein
26	1767	J3	0.025	1.31	Failed
27	1713	J4	0.018	−1.39	Collagen type I alpha 1
28	1769	J5	0.048	−1.59	Failed

**Table 4:** Information of 16 identified differential protein spots.

Number	Spot code	Protein name	Accession number	*t*-test *P* value	Average ratio	PI	MW	Peptide count	Protein Score	C.I.%	Overexpressed in
1	751	Alpha-actin	gi∣178027	0.00079	−1.92	5.23	42480	5	111	100	E
2	716	Heat shock protein 60	gi∣77702086	0.042	−3.17	5.7	61345.5	9	303	100	E
3	768	Desmin	gi∣55749932	0.033	−1.62	5.21	53560.2	31	916	100	E
4	974	Alpha-actin	gi∣4885049	0.0054	3.37	5.23	42334	9	207	100	C
5	1017	Alpha-actin	gi∣4501883	0.048	1.23	5.23	42381	15	579	100	C
6	1043	Alpha-actin	gi∣178067	0.001	−1.39	5.19	37125.3	8	130	100	E
7	991	Elongation factor Tu	gi∣704416	0.043	−1.4	7.7	49851.3	16	353	100	E
8	956	Alpha-actin	gi∣178027	0.0063	1.4	5.23	42480	15	579	100	C
9	1209	Tropomyosin alpha-1 chain	gi∣63252898	0.0044	1.34	4.69	32745.7	22	571	100	C
10	1164	PDZ and LIM domain protein 1	gi∣13994151	0.034	−1.53	6.56	36505.2	11	315	100	E
11	1234	Malate dehydrogenase	gi∣119620368	0.032	1.61	7.62	31920.5	7	159	100	C
12	1369	Proteasome subunit alpha type-1	gi∣13543551	0.017	−1.71	6.15	29864	6	232	100	E
13	1505	peroxiredoxin 6	gi∣4758638	0.048	−1.2	6	25133.2	12	427	100	E
14	1538	cysteine and glycine-rich protein 3	gi∣4502893	0.042	−1.33	8.89	21867.3	9	387	100	E
15	1754	CABC1 protein	gi∣120538499	0.0045	1.59	8.73	44563.7	3	117	100	C
16	1713	collagen type I alpha 1	gi∣119615036	0.018	−1.39	5.93	85144.5	9	87	99.947	E

E: Experimental Group; C: Control Group.

## Abbreviations

RHD: Rheumatic heart disease
ARF: Acute rheumatic fever
2D-DIGE: Two-dimensional difference gel electrophoresis
MALDI-TOF-MS: Matrix-assisted laser desorption ionization time-of-flight mass spectrometry
EF: Ejection fraction
LVEDD: Left ventricular end-diastolic diameter
HSP 60: Heat shock protein 60
MDH: Malate dehydrogenase
CABC1: Chaperone activity of bc1 complex homolog.
